# HCMV glycoprotein B is expressed in primary glioblastomas and enhances growth and invasiveness via PDGFR-alpha activation

**DOI:** 10.18632/oncotarget.1787

**Published:** 2014-03-12

**Authors:** Charles Cobbs, Sabeena Khan, Lisa Matlaf, Sean McAllister, Alex Zider, Garret Yount, Kenneth Rahlin, Lualhati Harkins, Vladimir Bezrookove, Eric Singer, Liliana Soroceanu

**Affiliations:** ^1^ California Pacific Medical Center Research Institute, San Francisco, CA; ^2^ The Swedish Neuroscience Institute, Ben and Catherine Ivy Brain Tumor Center, Seattle, WA, and; ^3^ University of Alabama at Birmingham, Birmingham AL

**Keywords:** Human Cytomegalovirus Glycoprotein B, glioblastoma, tumor invasiveness, Platelet Derived Growth Factor Receptor Alfa, slice invasion assay, time-lapse videomicroscopy

## Abstract

Our laboratory first demonstrated that human cytomegalovirus (HCMV) is associated with the most deadly form of primary brain tumor, glioblastoma (GBM). We showed that HCMV glycoprotein B (gB) mediates viral cellular entry via the receptor tyrosine kinase PDGFR-alpha (PDGFRα), resulting in activation of the PI3K/Akt pathway, a critical signaling axis gliomagenesis. Here, we investigated the effects of gB overexpression on glioma progression. We demonstrate that gB is endogenously expressed in primary GBM samples and show that ectopic gB expression in glioma cells induced sustained phosphorylation of PDGFRα, Akt, and Src. Recombinant gB protein and the whole virus enhanced invasion of primary glioblastoma cells into Matrigel and rat brain slices, and this effect was specifically inhibited by neutralizing antibodies to either gB or PDGFRα. Importantly, neutralizing antibodies to gB significantly inhibited the invasiveness of patient-derived HCMV-positive glioblastoma cells, suggesting that functional inhibition of this viral protein could hinder glioblastoma progression. gB overexpression promoted in vivo glioma growth and enhanced phosphor-Akt levels and tumor cell dispersal relative to controls. Taken together, our results demonstrate that HCMV gB promotes key hallmarks of glioblastoma and suggest that targeting gB may have therapeutic benefits for patients with HCMV -positive gliomas.

## INTRODUCTION

Human primary brain tumors (gliomas), including the most common and malignant type- glioblastoma (GBM) are extremely lethal cancers, with a median patient survival of~14 months. No etiological agent has been identified for gliomas. Malignant gliomas are highly invasive tumors, infiltrating the surrounding healthy brain tissue, which makes complete tumor removal by surgery unlikely. Our laboratory first reported that human cytomegalovirus (HCMV) gene products are expressed in over 90% of GBMs, while absent in the surrounding non-malignant brain tissue. These findings have been confirmed by several other groups [1-3]

To date, the role of HCMV proteins in the pathogenesis of malignant gliomas has not been elucidated. We recently discovered that activation of a receptor tyrosine kinase implicated in the pathology of gliomas, human platelet-derived growth factor alpha receptor (PDGFRα) is required for HCMV infection[4]. We determined that HCMV specifically binds and activates PDGFRα and downstream PI3K-Akt signaling in astro-glial and endothelial cells[4, 5]. We further determined that HCMV glycoprotein B (gB, the most abundant viral envelope glycoprotein) is the viral moiety that directly interacts with and tyrosine phosphorylates PDGFRα [4]. Indeed, recombinant HCMV gB (gB 680) induces activation of the oncogenic PI3-Akt pathway in glioma cells to a similar extent as does the genuine ligand, PDGF. We have previously shown that HCMV short term infection promotes cell survival and growth and enhances glioma cell haptotactic migration[5, 6].

The PDGFα-receptor gene is amplified in a subset of human glioblastoma [7, 8] suggesting that activation of PDGF receptor signaling confers a selective growth advantage in tumor growth. Evidence from mouse models of gliomas demonstrated that genetic alterations, such as loss of Ink4Arf locus in cooperation with exacerbated growth factor/growth factor receptor signaling in neural precursor cells can drive gliomagenesis [9, 10]. In vivo gene transfer of PDGF to neural precursor cells and astrocytes induces the formation of high grade gliomas in a dose dependent manner, suggesting that chronic activation of the PDGF receptors can promote proliferation of glial precursors and furthermore activate downstream signaling pathways (such as the PI3K-Akt axis) sufficient to drive tumorigenesis[11].

Given our preliminary data demonstrating that HCMV gB envelope glycoprotein could activate the PDGFRα, and induce downstream activation of the oncogenic PI3-K / AKT pathway, we sought to determine whether HCMV gB transcript and protein are endogenously expressed in human glioblastoma specimens and investigate the functional consequences of gB overexpression or stimulation using recombinant gB protein in glioma cells expressing PDGFRα. Using a mouse model of disease, we investigated the effects of gB overexpression on glioma growth and invasion *in vivo*. Our results delineate tumor promoting signaling pathways specifically activated by HCMV gB in glioblastoma and demonstrate that functional inhibition of the viral glycoprotein can impede tumor invasiveness.

## RESULTS

### HCMV promotes glioblastoma motility and invasion in a PDGFR-alpha dependent manner

**Figure 1:**
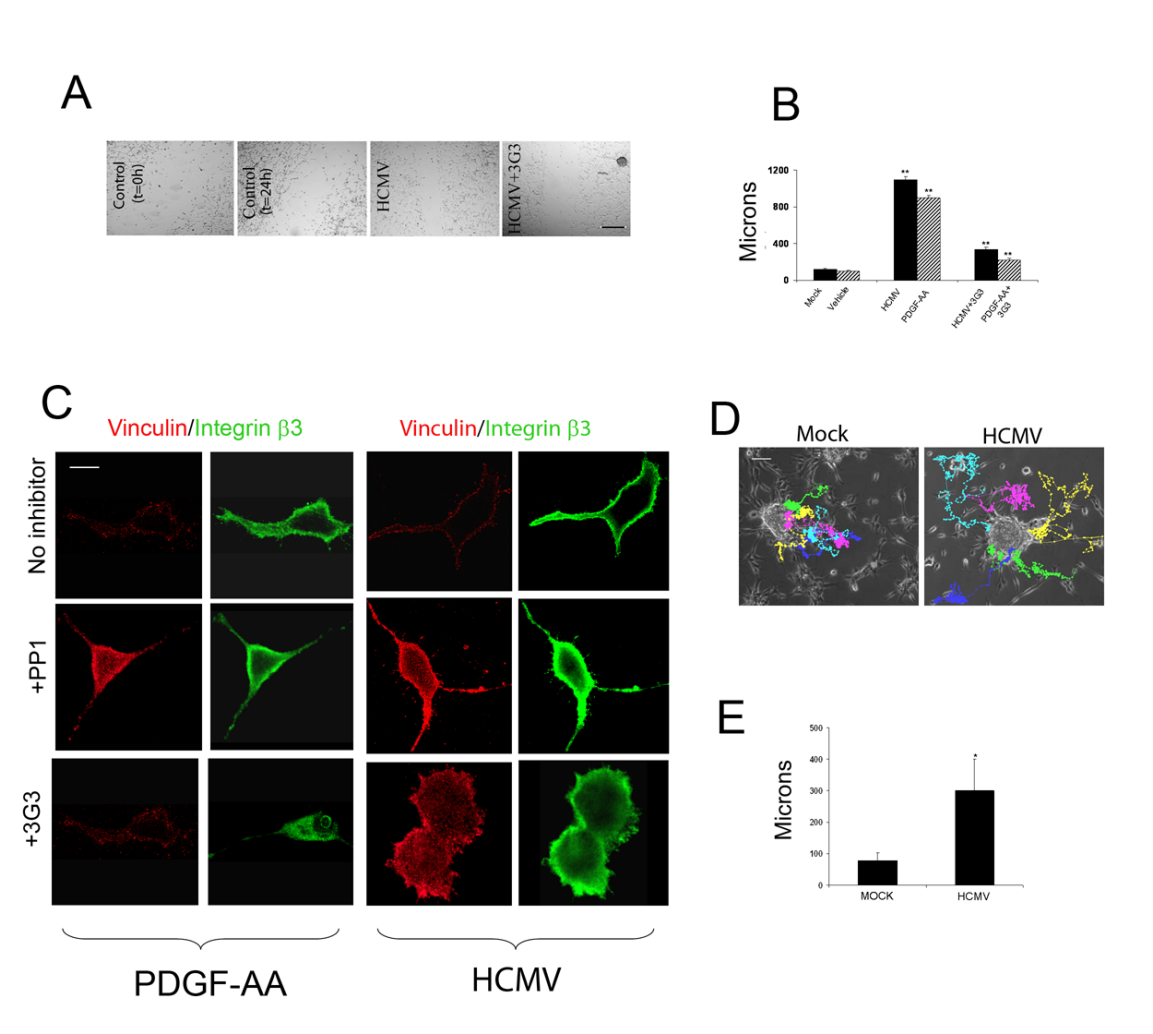
HCMV promotes glioma cell motility and enhances primary glioma stem cell migration A. Sixty thousand U87 cells/well were plated onto vitronectin-coated (5 ug/ml) coverslips and cultured overnight. Cells were then serum starved in DMEM and 1% BSA for 24h. The confluent monolayer was scratched with a 1000 ul pipette tip and photographed at 4x magnification under a light microscope immediately after scratching and 24h following the initiation of the “wound closure” process. Cells were pre-treated with blocking agents and stimulated with PDGF-AA, HCMV, or mock, as indicated. B. Quantification of the distance covered by the cells at the edge of the wound. Each condition was run in 6 wells of a 24 well plate and the experiment was repeated twice. *p<0.02, Student t-TEST. C. 30,000 U87 glioma cells were plated onto vitronectin-coated (5 ug/ml) coverslips and serum-starved overnight. Cells were treated with blocking reagents for 30 minutes, stimulated with PDGF-AA (20ng/ml), HCMV (MOI=1), or Mock for 10 minutes and processed for immunofluorescence. Cells were visualized at 60X on a Nikon confocal microscope. PDGF and HCMV treatment induced recruitment of the αvβ3 integrin to focal adhesions in cell cortex (upper rows) and this effect was inhibited by the Src inhibitor PP1 (2.5mM, middle row) and by the PDGFRα blocking antibody 3G3 (10ug/ml, lower panels). D. Tracking of GBM cell migration away from neurospheres growing on surface of cell culture plates under control conditions without HCMV (left panel) or after exposure to HCMV (right panel). Individual cells are indicated by distinct colors. Time indicated in hours. The average total displacement of GBM cells was increased by more than two-fold (unpaired t-test, p<0.001 ).

To directly compare the effects of whole virus HCMV (Towne, MOI=1) and the genuine ligand, PDGFAA on PDGFRα-mediated glioma cell migration, we used a previously described wound closure (scratch migration) assay [12]. HCMV induced increased migration of U87 glioma cells to a similar extend as PDGF-AA (20ng/ml) and this effect was significantly reverted by the co-incubation with the 3G3, a PDGFRα blocking antibody (Figure 1A-B). PDGF-AA has been previously shown to induce glioma cell invasion by activating PDGFRα in conjunction with recruitment of integrin αvβ3 to the focal adhesion cellular contacts [13, 14] a process dependent on activation of the Src oncogene. Since HCMV can activate PDGFRα to a similar extent as the natural ligand, we set out to investigate the effects of HCMV on glioma cell stress fibers, which support enhanced tumor cell motility. U87 cells plated on vitronectin coated glass slides were stimulated with PDGF-AA or HCMV in the presence or absence of 3G3 blocking antibody or PP1 Src inhibitor [13]. Twenty four hours later cells were processed using double immunofluorescence to detect vinculin and integrin β3 [13]. Confocal microscopy of glioma cells revealed that similarly to PDGF-AA, HCMV induced stress fiber reorganization as demonstrated by vinculin and β3 integrin co-localization and this process was dependent on PDGFRα and Src activation, since it was blocked by pretreatment with 3G3 (10 μg/ml) or PP1 (2.5mM, Figure 1C). To investigate tumor cell motility in a three dimensional environment, we used primary –derived glioblastoma neurospheres cultured in matrigel in the presence of vehicle or HCMV (Towne, MOI=1) and followed the motility of individual GBM cells using time-lapse video microscopy. Based on qualitative assessment of time-lapse videos, there was an apparent increase in the GBM cell migration away from the neurospheres into the matrigel (data not shown). Quantitative tracking of neurospheres growing in two dimensional cultures confirmed this dispersal from the neuroshere by individual cells (Figure 1D). The average total displacement of GBM cells was approximately two-fold greater in experiments with HCMV (177 μm+/− 22 um SE) compared to without HCMV (84 μm+/− 10 um SE) over a 48 hour period (Figure 1E, p=0.001, unpaired t-TEST).

### HCMV glycoprotein B mRNA and protein and human PDGFR-alpha are co-expressed in primary glioblastoma tissue and cells

**Figure 2:**
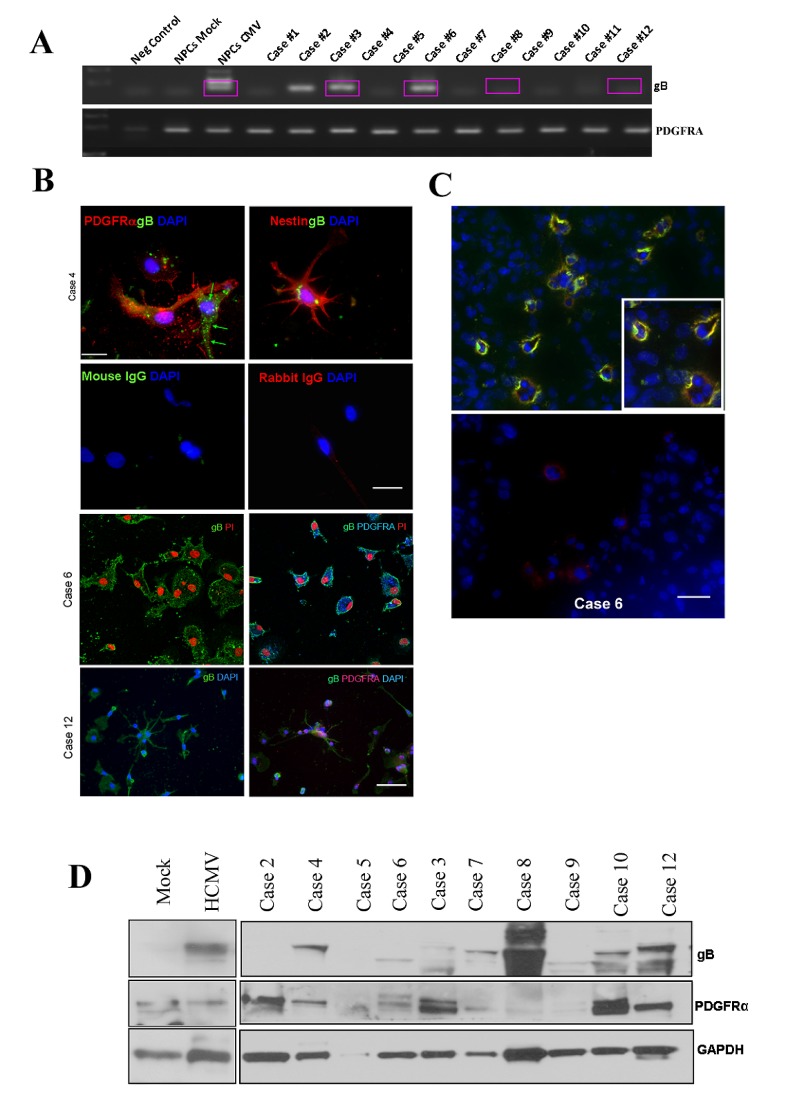
HCMV glycoprotein B mRNA and protein are endogenously expressed in human glioblastoma tissues and co-localize with PDGFRα in situ A. RT-PCR products obtained using primers specific for gB (upper panel) and PDGFRα were separated on an agarose gel. All RT-PCR products were sequenced; boxed lanes indicate samples where the identity of gB was confirmed by sequencing. B. Primary –derived GBM cells were cultured on coverslips and processed for immunofluorescence as previously described (ref). Upper 4 panels show one case where gB (green fluorescence) and PDGFRα (red fluorescence) are expressed in adjacent tumor cells. Nestin staining is shown in the upper right panel. Non-specific staining was controlled for by using isotype matched primary antibodies (second row). Bar= 75μm.Third and fourth rows show two additional GBM primary cell samples stained for gB (left panels) or gB and PDGFRα (right panels). Nuclei are stained with DAPI (blue) or PI (red). Bar= 100μm. C. Immunofluorescence analysis of frozen GBM tissue using gB and PDGFRα antibodies. Upper panel shows examples of tumor cells positive for both antigens. Lower panel displays a negative control. Nuclei were stained using DAPI. Bar=50μm. D. gB and PDGFDRα detection using Western blot analysis of whole tumor tissues from several CPMC GBM cases. The blot was stripped and re-probed for GAPDH.

To establish clinical relevance for our investigation, we next measured the expression levels of HCMV gB in primary glioblastoma tissues and cells. Nested RT-PCR analysis of several glioblastoma samples show the presence of gB transcript endogenously expressed in glioma tissue (Figure 2A). All RT-PCR reaction products were sequenced to confirm the identity of the transcript and exclude the possibility of contamination with laboratory viral strains (sequence alignment is provided as supplementary information, Supplementary Figure 1). PDGFRα mRNA was detected in all tested samples. Using double immunofluorescence, we next evaluated expression of HCMV gB protein in primary GBM cells obtained from acutely dissociated tumor tissues. Cells were processed for gB and PDGFRα immunostaining as shown in Figure 2B. We found several primary-derived GBM cultures expressed gB and PDGFRα in the same cells; in some cases the two proteins were expressed on the surface of adjacent cells, supporting the notion that both autocrine and paracrine signaling via gB-PDGFRα may occur in human GBMs in situ. Using double immunofluorescence, we interrogated several flash frozen GBM tissue samples for the presence of gB and PDGFRα proteins. As shown in a representative example (Figure 2C), HCMV gB and PDGFRα are co-localized in human GBM tissues, in situ. This is significant, since it suggests that gB can engage PDGFRα to promote oncogenic signaling.

To corroborate these data, we generated protein lysates from some of the same tissue samples analyzed by RT-PCR and used western blot analysis to detect both gB and PDGFRα. As shown in Figure 2D, gB was detected in several cases; in some samples several gB isoforms (as shown by different molecular weight proteins) were present. This is not unexpected, since HCMV gB has been shown to be modified at the post-translational level in infected cells [15]. PDGFRα was expressed at various levels in the tested samples. The blot was stripped and probed for GAPDH to demonstrate equivalent loading (Figure 2D). Taken together, these results demonstrate that gB and PDGFR co-expression is a frequent occurrence in human GBMs.

### HCMV gB overexpression induces activation of the endogenous PDGFR- AKT pathway, which promotes glioma cell invasion

**Figure 3:**
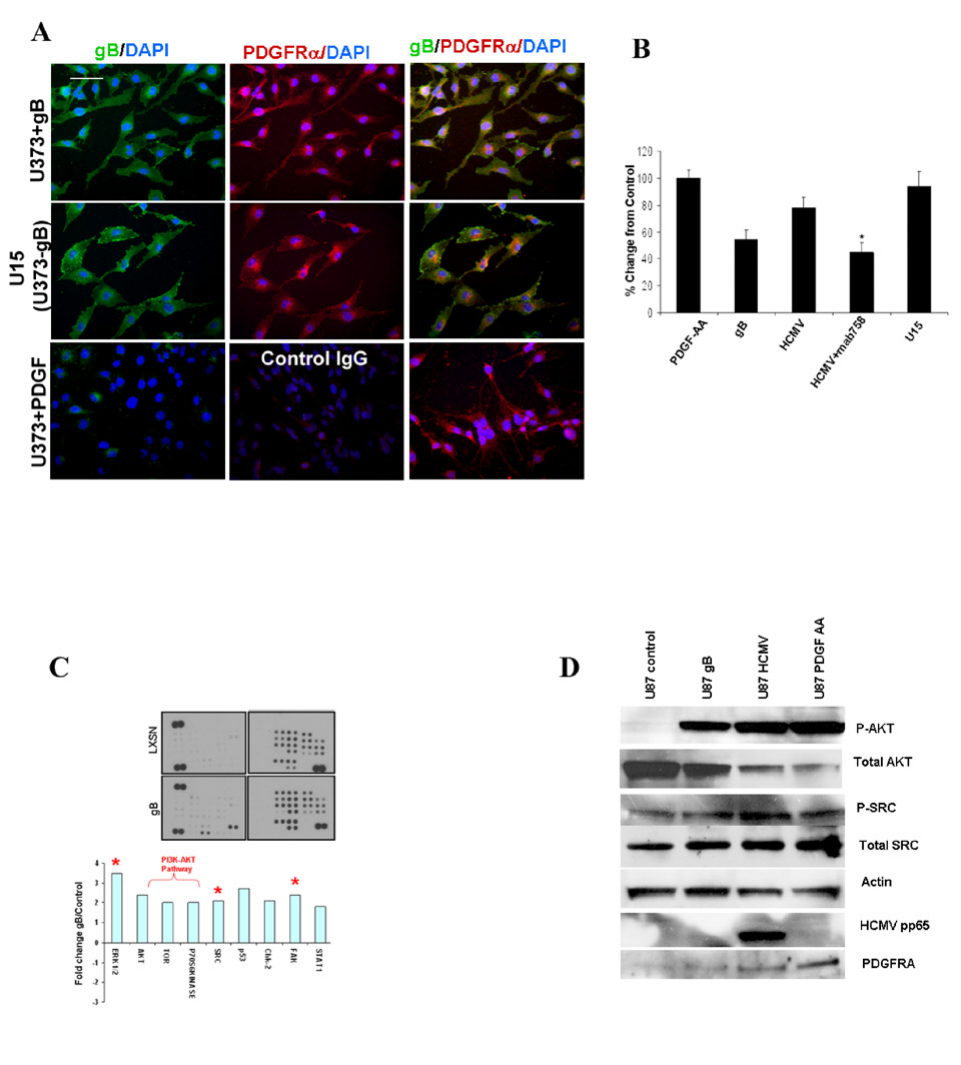
HCMV gB ectopic expression induces activation of the phospho-PDGFRα-PI3K-AKT pathway in human glioblastoma cells A.U373 glioma cells grown in serum free conditions (lower panels) stimulated with PDGF-AA (5 ng/ml, 20 min), stably expressing gB (middle panels, U15) or stimulated with recombinant HCMV gB were immunostained for gB (green fluorescence)and phospho-PDGFRα (red fluorescence). Nuclei counterstained with DAPI. gB expressing U15 cells (U373 stably expressing gB) exhibit activation of PDGFRα in the absence of extrinsic stimuli. gB and p-PDGFRα are co-localized at the leading edge of the U15 cells (arrows). Bar= 100μm. B. Quantification of matrigel invasion of U373 glioma and U15 cells stimulated as indicated. Each condition was run in quadruplicate and the experiment was repeated twice. All cells which migrated through Matrigel were stained and counted. A representative experiment is shown. * p=0.01, student t-TEST. C. Glioma cells stably expressing recombinant gB and control (LXSN) were interrogated using a phosphor-kinase proteome profiler array. Densitometry measurements were used to quantify the results, shown in the lower panel. Expression of marked phosphor-proteins were confirmed using Western blot. D. U87 cells were stimulated as shown and processed for western blot using the indicated antibodies.

To investigate the effects of gB on endogenous PDGFRα-mediated signaling, we used the U373 astrocytoma cell line stably expressing gB – U15 (a gift from Dr. Lenore Pereira, UCSF, [16]) as well as the parental U373 line and measured phosphorylation levels of PDGFRα in control conditions and after stimulation with PDGF-AA or gB. All cells were maintained in serum free media for receptor phosphorylation studies. Figure 3A shows that gB stimulation of U373 induces phospho-PDGFRα to a similar extent as the genuine ligand, PDGF-AA (upper and lower rows). Interestingly, U15 cells showed constitutive phosphorylation of PDGFRα in the absence of any external stimulus and this effect could not be further enhanced by adding PDGF-AA ligand (not shown). U15 cells, but not the control U373 glioma cells, displayed cytoskeleton features such as a fan-like lamellipodium, consistent with a migratory tumor cell phenotype. Using a highly sensitive cell based ELISA [4], phosphorylation levels of PDGFR were measured in U373 stimulated with recombinant gB or whole virus in the presence or absence of MAB758, a gB blocking antibody[17]. HCMV gB induced a 50% increase in PDGFRα phosphorylation as compared to 100% induced by PDGF-AA (positive control). Blocking gB using the MAB 758 antibody significantly inhibited HCMV-induced receptor phosphorylation (Figure 3B). Remarkably, U15 cells displayed >80% phosphorylation of PDGFRα in baseline conditions, which is in agreement with the immunofluorescence analysis (Figure 3A-B). U15 cells exhibited enhanced matrigel invasion compared to U373 controls, which could be inhibited by gB blocking antibody (Supplementary Figure 3). To further investigate gB-induced tyrosine kinase mediated signaling in glioma cells, we used a retrovirus based system to stably transduce U87 glioma cells with HCMV gB. Following selection in G418-containing media, gB expression was verified by western blot and immunofluorescence analyses (Supplementary figure 3 and data not shown). Matrigel invasion of gB expressing U87 cells was significantly enhanced compared to control LXSN-expressing glioma cells (Supplementary Figure 3). U87 cells expressing gB and controls were serum starved for 48h and cell lysates hybridized to a non-receptor tyrosine kinase antibody array [18]. Quantitative analysis demonstrated that sustained expression of gB induced significant activation of the PI3K-AKT, p-ERK, Src and FAK pathways in glioma cells as compared to control (Figure 3C, lower panel). To validate these results we used western blot analysis of U87 cells stimulated with recombinant gB or HCMV. Our data shows that gB induced activation of the AKT and Src pathways (Figure 3D). Using primary-derived HCMV negative glioma stem-like cells (GSC, line 4121), we found that HCMV gB induced activation of the AKT pathway via phosphorylation of PDGFRα (Supplementary Figure 4). These results suggest that gB overexpression can promote oncogenic signaling in PDGFRα expressing GSC, driving gliomagenesis.

### Functional blocking of HCMV gB inhibits invasion of primary glioblastoma cells

**Figure 4:**
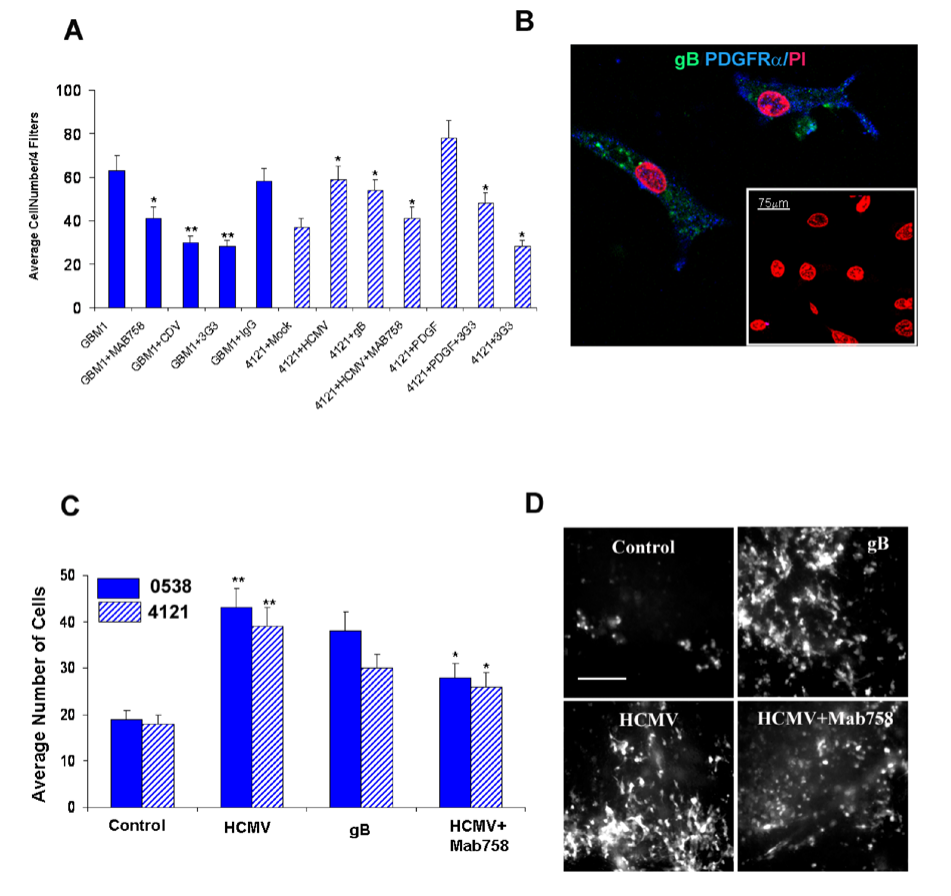
HCMV gB promotes glioblastoma cell invasion into matrigel and brain tissue slices A. Matrigel invasion assays were performed using a primary GBM (GBM 1) treated as indicated. Each condition was run in quadruplicate and quantification was done using 4 filters/condition. Average number of cells are displayed (solid bars). Primary GSC line 4121 was subjected to Matrigel invasion assay following various treatments, as indicated. Each condition was run in quadruplicate and this experiment was repeated twice. Average number of cells counted in 4 filters from a representative experiment are shown. * p<0.02, student t-test. B. Immunofuorescence analysis of primary glioma cells (GBM1) demonstrate endogenous expression of gB (green fluorescence) and PDGFRα (blue fluorescence) 48h following initial culturing. Nuclei are counterstained with propidium iodide. Bar= 75μm. C. Quantification of slice invasion assays using two glioma stem cell lines (5938, solid bars and 4121, hashed bars) treated as indicated. Each condition was run in quadruplicate and the experiment was repeated three times. * p= 0.003, student t-test. D. Representative photomicrographs of GFP-labeled GSC cells, treated as indicated, following 48h invasion through a 2mm rat brain slice captured using a Nikon inverted microscope fitted with a camera. Bar= 200μm.

We next focused on functional assays to assess the role of gB in modulating glioblastoma cell invasiveness. Matrigel invasion assays were conducted as previously described by our group [18, 19]. Since we found that gB induced a significant increase in glioma cell invasion compared to controls (Supplementary Figures 2-3), we sought to use patient-derived primary GBM cells (confirmed HCMV gB positive) and investigate the effects of gB loss of function in an endogenously infected tumor. Primary GBM cells were subjected to Matrigel invasion assays within 48 hours of initial culturing (Figure 4A-B). Twenty four hours pre-treatment with either PDGFRα blocking antibody (3G3 from Imclone Inc [4]), gB blocking antibody (MAB 758), or the antiviral drug Cidofovir (40μM) inhibited the baseline invasiveness of the primary GBM cells, as shown in Figure 4A (solid bars). These data suggest that HCMV gB is a potent inducer of cell invasion in endogenously infected GBMs. Conversely, we used a confirmed HCMV negative primary-derived GSC line (4121) to measure effects of HCMV and gB on tumor cell invasion into Matrigel. Our data shows that HCMV enhances GSC invasion in a gB and PDGFR dependent manner (Figure 4A, hashed bars).

To more closely replicate the brain tissue architecture, we next used an organotypic neonatal rat brain slice invasion assay [18] to assess the role of HCMV gB in modulating GBM invasiveness. Using two primary GSC lines labeled with green fluorescence protein (GFP), using quantitative fluorescence microscopy, we show that HCMV promotes GBM slice invasion in a gB-dependent manner (Figure 4C-D).

### HCMV gB promotes cell cycle progression in human glioma cells

**Figure 5:**
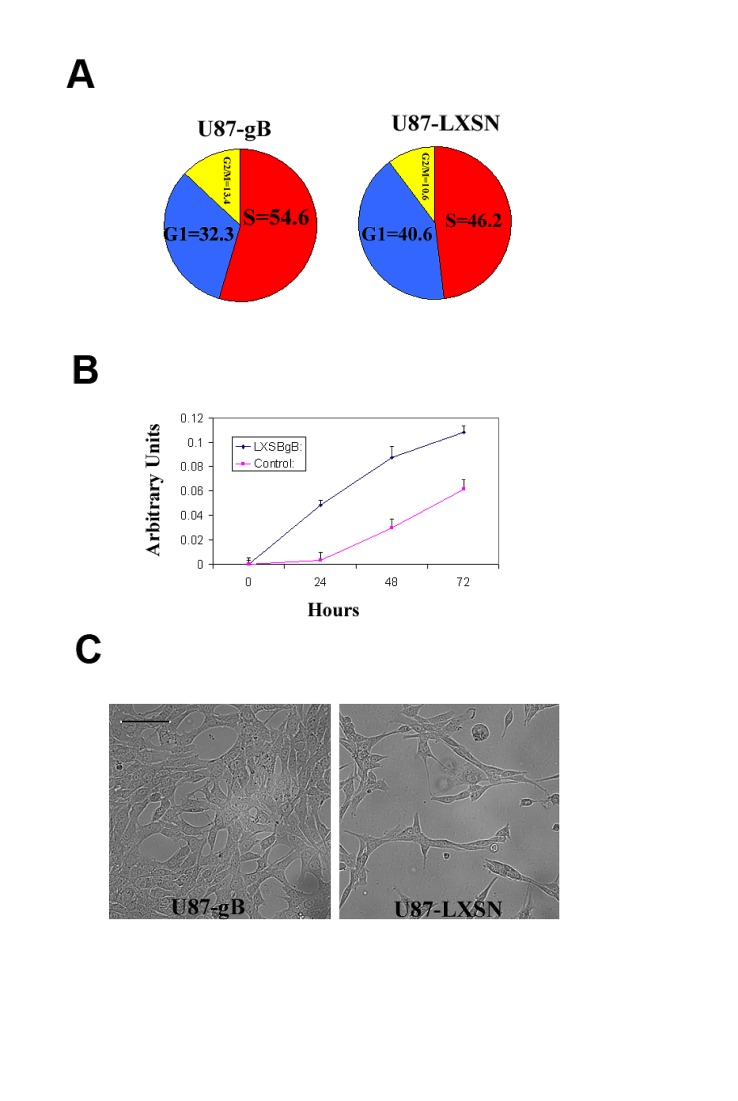
HCMV gB promotes glioma cell proliferation A. Pie chart representation of the cell cycle FACS analysis for U87-gB and U87-LXSN cells following 48h of serum starvation. 10,000 events were analyzed for each condition. The experiment was repeated three times. A representative result is shown; p=0.03, Student t-Test. B. MTT cell survival assay used to compare the baseline growth rates of U87-gB and U87-LXSN cells for 72h following 24h of serum starvation. Each condition was run in 6 wells of a 96 well plate. The experiment was repeated twice and a representative result is shown p = 0.01, student t- Test. C. Representative photomicrographs of U87-gB and U87-LXSN cells captured at 48 h following serum deprivation. Initial cell density was 5000 cells/well. Bar= 75μm.

Proteome profiling of gB expressing and control glioma cells demonstrated activation of several pathways critical for both tumor cell invasion as well as tumor cell survival and growth, including phosphor-ERK and phosphor-AKT. We therefore sought to measure the effects of ectopic gB expression on the proliferation of glioma cells. Cell cycle analyses showed an 8.3 % (+/− 0.6) increase in the S phase in gB expressing U87 cells as compared to control (Figure 5 A). MTT viability assays showed that gB expression conferred a survival advantage to glioma cells as compared to control (Figure 5 B-C), suggesting that HCMV gB promotes glioma aggressiveness by activating both tumor cell invasion as well as promoting glioma cell survival and growth.

### HCMV gB enhances glioma aggressiveness in vivo

**Figure 6:**
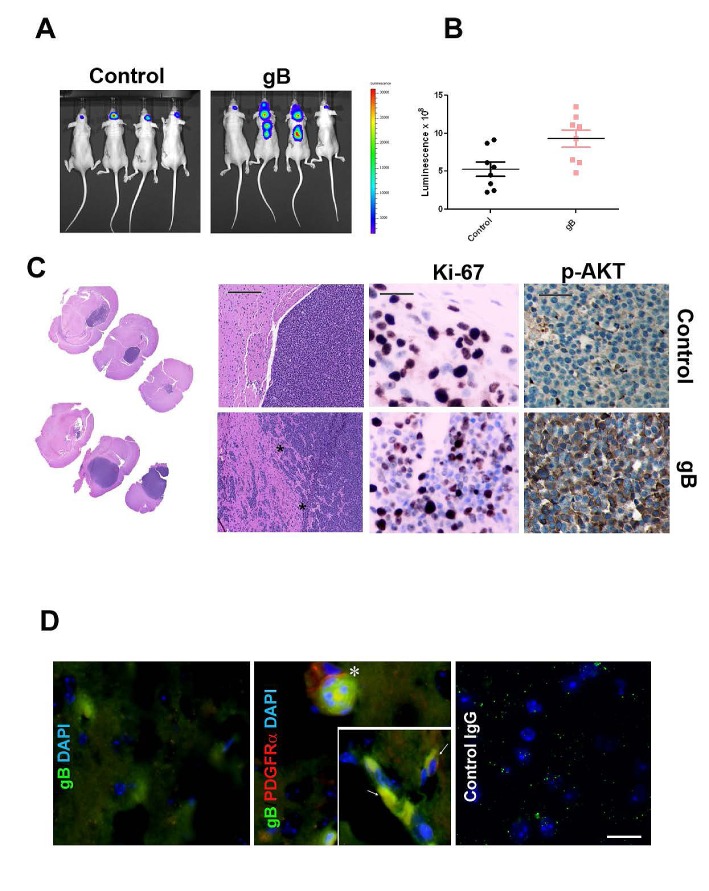
HCMV gB enhances glioblastoma aggressiveness *in vivo* A. Representative bioluminescence images of nude mice bearing U87-Luc intracranial gliomas transduced with gB or control PLXSN vector. Images were acquired 28 days following implantation of 300,000 cells. B. Bioluminescence imaging measurements at day 28 post tumor implantation. C. H&E staining of U87-control (upper left panels) and U87-gB expressing xenograft tissues (lower left panels) at two different levels of magnification (5X and 20X). Bar= 200μm. Middle panels: Ki67 immunostaining of tumor tissue sections. Right panels show IHC detection of p-AKT in consecutive sections from the same tissue samples. Representative images are shown Bar=75μm. D. Double immunofluorescence was used to detect HCMV gB (green fluorescence) and phospho-PDGFRα (red fluorescence) in U87-gB expressing tumor tissue. Inset shows a higher magnification of gB expressing glioma cells which also express p-PDGFRα. Nuclei are stained with DAPI. Bar=100μm.

To investigate the effects of ectopic gB expression on gliomagenesis in vivo, we used Luciferase labeled U87-gB and U87-LXSN glioma cells to generate intracranial xenograft tumors in nude mice (12 mice/group). We used bioluminescence imaging to monitor the growth rate of tumors in real time. Four weeks following implantation of 250,000 cells, gB expressing U87-derived gliomas displayed significantly more invasive growth patterns compared to the control U87 tumors, which correlated with the higher luminescence signal (Figure 6A-B).

Tissue sections from U87 tumor xenografts were processed by immunohistochemistry. Hematoxylin and eosin staining (Figure 6C, left panels) demonstrates the invasive growth pattern of the gB expressing tumors as compared to the well defined tumor margin in the control tumors. U87-gB expressing tumors show ~15% increase in Ki-67 staining (Supplementary Figure 5) and significantly augmented phosphor-AKT levels compared to control U87 tumors (Figure 6 C, middle and right panels). These results suggest that gB expressing gliomas are significantly more aggressive compared to controls, in agreement with our data from tissue culture experiments. Flash frozen xenograft tissue sections harvested from U87-gB expressing tumors were processed for double immunofluorescence to detect PDGFRα and gB. As shown in Figure 6D, gB and PDGFRα are found co-expressed in the same or adjacent tumor cells in situ (arrows), suggesting that autocrine or paracrine signaling initiated by gB, likely activated PDGFRα and downstream tumor promoting pathways (p-AKT), leading to enhanced glioma aggressiveness in this mouse model of disease.

## DISCUSSION

Tumor cell invasion into the surrounding healthy brain tissue renders gliomas impossible to completely remove by surgery, and thus leads to tumor recurrence, which is usually fatal. Therefore, understanding the mechanism underlying glioma invasiveness is critical for designing improved therapeutic approaches. To date, several laboratories have shown that HCMV and specifically HCMV glycoprotein B is found in a majority of human primary glioblastomas (this study and [2, 20]). Since gB can act as a PDGFRα ligand, and signaling via this receptor tyrosine kinase drives a subset of human glioblastomas, we sought to investigate the specific pathways impacted by HCMV gB signaling via PDGFRα in human brain tumors. Using several approaches to overexpress gB in glioma cells, we intended to simulate chronic activation of the PDGFRα, which has been linked to disease progression in glioblastoma [11]. Our results show that gB induces p-PDGFRα and downstream signaling partners such as AKT, Src, FAK, which promote tumor cell survival and motility. This stimulation could be partially reverted by using either gB or PDGFRα blocking antibodies, demonstrating specificity of the observed effect.

The use of time-lapse video microscopy allowed us to visualize increased cell migration away from growing primary GBM neurospheres, in real-time, following exposure to HCMV. These results reinforce the potential role of HCMV in promoting the invasiveness of GBM cells since migration away from the originating neurosphere is a possible indication of a tumor's capacity to invade surrounding, healthy tissues.

We show that human glioblastomas endogenously express gB and PDGFRα, often in the same cells, which suggests that this growth promoting signaling pathway may indeed contribute to the invasive phenotype of HCMV positive GBMs. Furthermore, the invasive behavior of freshly dissociated primary GBM cells, confirmed gB positive, could be significantly inhibited by treatment with gB blocking antibodies. These data suggest that targeting gB may have therapeutic benefits.

Using a mouse model of disease, we show that gB expressing intracranial gliomas grow more aggressively compared to controls and activate critical tumor-promoting pathways (e.g., p-AKT). Taken together, our data supports an important role for HCMV gB signaling via PDGFRα to promote glioma pathogenesis.

Currently, it is unclear whether HCMV infection is an initiating event or a secondary event in the pathobiology of glioblastoma. Clearly, sustained activation of PDGFRα signaling can drive early neoplastic changes in glioma development [11]and PDGFRα plays a major role in glioblastoma pathogenesis. Since HCMV gB protein expression has been detected in a majority of glioblastomas in vivo by several groups [2, 21, 22], and we show that gB functions essentially as an authentic PDGFR ligand, our data indicate that HCMV is exerting autocrine and paracrine oncogenic effects in vivo.

Importantly, these findings immediately suggest that antiviral strategies that inhibit HCMV viral gene products could benefit patients by decreasing the oncogenic properties of the gB- PDGFRα signaling cascade and potentially other viral-mediated oncogenic pathways. We have recently shown that Cidofovir inhibits glioblastoma progression in vivo using a mouse model of disease [23]and a small clinical trial report showed that adjuvant treatment with Valganciclovir- which blocks late HCMV viral gene expression (such as gB)- improved survival in glioblastoma patients [24]. These results emphasize the need for more in-depth investigation of the potential of antiviral drugs as anti-glioma agents.

## MATERIALS AND METHODS

### Ethics Statement

All human brain tissues (including glioblastoma samples processed as described below) used in these studies were obtained from the CPMC Neurosurgery Department, under an IRB approved protocol (Protocol # 25.125-1). All patients provided written consent stating that they allowed for their tumor samples to be used for basic research. The California Pacific Medical Center Institutional Review Board approved the tissue collection protocol, including the patient consent forms (Current IRB Assurance NO: FWA00000921). Samples have been de-identified before being processed, to protect patient privacy

### Primary GBM tissue sample and neurosphere growth assays

Tissue samples were obtained during surgery from patients diagnosed with GBM using an IRB-approved protocol. They were then subjected to enzymatic digest, mechanically dissociated, and cultured as neurospheres as previously described by our group [6].

### RNA and protein isolation from whole tissue

Brain tissue was homogenized and lysed in 1 mL QIAzol reagent (Qiagen) using a TissueRuptor homogenizer (Qiagen). RNA was then chloroform extracted and purified using the RNeasy lipid tissue mini kit (Qiagen). The quality of the RNA was verified by spectrometry and visualization of ribosomal RNA bands on an agarose gel. DNA was precipitated from the remaining interphase and organic phase with 75% ethanol, and the protein in the supernatant was then isopropanol precipitated, denatured with 0.3M guanidine hydrochloride, and resuspended in 1% SDS.

### PCR Analysis

For standard end-point PCR experiments, 1 ug of each RNA was reverse transcribed into cDNA using the iScript cDNA synthesis kit (BioRad) or the SuperScript II kit (Invitrogen) and PCR amplified using the Taq PCR core kit (Qiagen) with an input of 50 ng of cDNA for each experimental sample and water only for the negative control. Control RNA from fetal brain and adult normal cortex were commercially obtained (BioChain). The primers used for nested RT-PCR detection of gB were:

gBF-external 5'- TCCAACACCCACAGTACCC;

gBR-external5'- CGGAAACGATGGTGTAGTTCG-3';

gBF-internal CCGCCCGCCCCGCGCCCGCCGCGGCAG CACCTGGCT-3';

gBR-internal5'- GTAAACCACATCACCCGTGGA-3';

PDGFRA was detected using the following primers:

5'-CTAATCCTCTGCCAGCTTTC-3' and 5'-TCACTTCCAAGACCGTCAC-3

### Viruses

Retroviruses pLXSN and pLSNX-gB were transfected into PT67 packaging cell lines and supernatants were harvested at 32 degrees and used to transduce U87 cells with 6 μg/mL polybrene. U87 cells were selected with 500 μg/mL G418 for 1 week and then G418 was removed. All HCMV infections were performed at an MOI of 1 with either the laboratory strain Towne (ATCC) or the clinical strain TR (gift from Dr. Lee Fortunato, University of Idaho). Presence of the clinical cassette region UL b' in TR was confirmed by PCR analysis after propagation.

### Immunohistochemistry and Immunofluorescence

Slides were baked and processed for immunohistochemistry and immunofluorescence as described previously described by our laboratory [19, 23, 25]. Briefly, slides were de-parafinized through a series of xylenes and ethanol, followed by antigen retrieval using Citra Plus solution (Biogenex). Slides were incubated overnight (4°C, in a humidified chamber) with primary antibodies or the mixtures of antibody and blocking peptide. Signal was detected using the “Super Sensitive Polymer-HRP” detection system (Biogenex), according to the manufacturer's instructions. Slides were counterstained with hematoxylin and de-hydrated using ethanol/xylenes. For integrin and vinculin detection, cells were treated with blocking reagents for 30 minutes (or fresh media), stimulated with PDGF-AA, HCMV, or Mock for 10 minutes. Cells were fixed in 4% buffered paraformaldehyde, permeabilized with 0.3% Triton X-100, blocked with TBS-BSA, and reacted serially with each set of primary and secondary antibodies. Primary antibodies used were anti-vinculin (Sigma, 1/500) followed by anti-mouse –Alexa 488 (1/3,000, Molecular Probes) and anti- β3 integrin (Chemicon, 1/400), followed by anti- rabbit-Alexa 568 (1/4,000, Molecular Probes). Cells were grown on glass slides in 24-well tissue culture dishes and treated as indicated. Prior to staining, cells were rinsed with PBS, fixed for 10 minutes in cold methanol, and then blocked for 30 minutes with protein free blocking buffer (Thermo Fisher). Primary antibodies were used as indicated, and the following secondary antibodies were used at a dilution of 1:1000: AlexaFluor488 anti-mouse IgG, AlexaFluor647 anti-rabbit IgG, and AlexaFluor647 anti-goat IgG (Invitrogen). Cells and were visualized using a Nikon Eclipse C1 Confocal microscope (Nikon TE2000-U) fitted with a “Cool Snap” Photometrix camera (Roper Scientific). Images were acquired using EZ-C1 v2.20 software and further processed using Adobe Photoshop CS4.

For cell cycle analyses, glioma cells engineered to express gB or the control LXSN vector were pulsed with 10 *μ*mol/L BrdU (Sigma) for 120 minutes prior to harvesting and fixation in 70% ethanol. Cells were subsequently denatured in 2 mol/L HCl and stained with anti-BrdU monoclonal antibody (Santa Cruz Biotechnology, Santa Cruz, CA) followed by FITC-conjugated secondary anti-mouse IgG (Molecular Probes/Invitrogen). After counter-staining with propidium iodide solution (10 *μ*mol/L) cells were analyzed by flow cytometry, as described [6].

### Organotypic (ex-vivo) invasion brain slice assay

1 mm coronal rat brain slices spanning from -0.94 to 1.98 of bregma were created from postnatal day three rat pups using a precision brain slicer and razors. The slices were then suspended in neurobasal (NB) complete media until ready for use. Slices were next transferred to millicell 8 μm pore size polyethylene terephthalate (PET) culture insert (Millipore) which was suspended in a 12-well plate containing 1.2 ml NB complete media in each well. The slices were left for 30 min to settle while the GBM cells were prepared. GFP-labeled primary GBM cells were resuspended at the appropriate concentration in NB complete media MAB 758 gB blocking antibody, 3G3 antibody or isotype specific control IgG (2μg/ml). The inserts with the slices were next transferred to a new 12-well plate containing 1.2 ml conditioned media made from fibroblast cultures. and 1x105 GFP-labeled cells were next placed on top of the brain slice by slowly pipetting a volume of 50μl. The cells were allowed to settle on top of the slice for one hour in the incubator. After one hour, an additional 150μl of medium was added around the slice to bathe the tissue. After three days, the slice was fixed in 2.5% glutaraldehyde for 15 min, rinsed with PBS, and then brightly GFP-labeled cells on the bottom of the slice were visualized and counted using an inverted fluorescence Nikon Eclipse TE2000-E microscope.

Time-lapse video microscopy was used to monitor cell movement. To facilitate accurate tracking, quantitative analyses were done on cells grown on the surface of cell culture plate without Matrigel. Cell culture plates were transferred to the microscope equipped with an environmental chamber that maintains routine incubation parameters (37°C, 5% CO_2_) using digital controllers. Phase contrast images from five (5) fields for each well were acquired at a magnification of 10x using a high-resolution, digital camera (ORCA C4742-80-12AG; Hamamatsu, Hamamatsu City, Japan) at 180-second intervals. Volocity Acquisition software (Improvision, Lexington, MA) controlled the camera and stage movements. Five (5) cells from each field were tracked using a mouse-based tracking program. Selection criteria for these cells were such that each starting point began at the edge of the neurosphere and clear tracking was available throughout the duration of the video. Final displacement for each tracked cell was determined accounting for neurosphere circumference and averages and standard errors were calculated for each condition.

### Xenograft intracranial model of GBM

All animal studies were carried out in accordance with NIH guidelines involving experimental neoplasia and the institutional (CPMCRI) approved IACUC protocol. Tumors were generated in female athymic *nu/nu* mice by the intracranial injection of 0.3x10^6^ LXSN- or gB expressing U87-Luc cells, as previously described by our group. Animals were removed from the study when they demonstrated any single sign indicative of significant tumor burden development, including hunched back, sustained decreased general activity, or a significant decrease in weight. Mice were imaged once weekly 15 minutes following Luciferin administration (15mg/kg), using an Ivis Lumina Instrument, which captured BLI images and corresponding measurements.

### Statistical analyses

Significant differences were determined using ANOVA or the unpaired Student's t-test, where suitable. Bonferroni-Dunn post-hoc analyses were conducted when appropriate. P values <0.05 defined statistical significance.

## SUPPLEMENTARY FIGURES


