# Supplementary material for: HCMV glycoprotein B is expressed in primary glioblastomas and enhances growth and invasiveness via PDGFR-alpha activation
Source: Oncotarget. 2014 Mar 12;5(4):1091–100. doi: 10.18632/oncotarget.1787 (PMC4011586; doi:10.18632/oncotarget.1787)
Supplement: Supplementary file 1 [file oncotarget-05-1091-s001.pdf]

HCMV glycoprotein B is expressed in primary glioblastomas and enhances growth and invasiveness via PDGFR-alpha activation

Charles Cobbs[1,2*], Sabeena Khan[1*], Lisa Matlaf[1], Sean McAllister[1], Alex Zider[1], Garret Yount[1], Kenneth Rahlin[1], Lualhati Harkins[3], Vladimir Bezrookove[1], Eric Singer[1], and Liliana Soroceanu[1]

SUPPLEMENTARY INFORMAITION

This file includes supplementary Figures 1-5 and legends.

CAACGGAACCAATCGCAATGCCAGCTACTTTGGAGAAAACGCCGACAAGTTTTTCATTTTCCCGA
CAACGGAACCAATCGCAATACCAGCTACTTTGGGGAAAATGCCGATAAGTTTTTCATTTTCCCGA
CAACGGAACTAATCGCAATGCCAGCTATTTTGGAGAAAACGCCGACAAGTTTTTCATTTTCCCGA
CGGCGGAACCAATCGCAATGCCGGCTACTTTGGATAAAACGCCTACAAGTTTTTCATTTTCCCGA
CGNCGGAACCNATCGCN TGCCTNCTACTTTGGAGAAAACGCCGACGAGTTTTTCATTTTCCCGA
TCTACGCGGANNAATCNTNNGTCGGNCTTTGNANAAAACGCCGACNAGTTTTTCATTTTCCCCA

ACTACACCATCGTTTCCG	**AD169**
ACTACACCATCGTTTCCG	**Merlin**
ACTACACCATCGTTTCCG	**TR**
ACTACACCATCGTTTCCG	**CPMC CASE 4**
ACTACACCGGCGGTTCCG	**CPMC CASE 6**
ACTACACCNTCGTTTCCG	**CPMC CASE 12**

Supplementary Figure 1. Human Cytomegalovirus Glycoprotien B Sequence Comparison. Sequence alignment for nested RT-PCR gB products obtained from three CPMC cases (shown in Figure 2A) and three published HCMV strains.

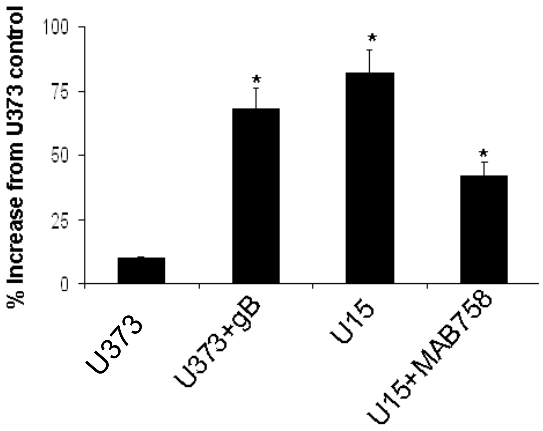

Supplementary Figure 2. **Stable gB expression enhances invasiveness of glioblastoma cells**. Matrigel invasion assays were performed using the U373 wild type cells and the gB stable expressing derivative (U15) in the presence of recombinant gB or MAB758 gB blocking antibody. Four filters/condition were used and the experiment was repeated twice. A representative result is shown. Results are expressed as percentage change from untreated U373 cells. Student T-test was used to assess significance. *p=0.001 for U373+gB vs U373, p=0.0003 for U15 vs U373 and p=0.01 for U15+MAB758 vs U15.

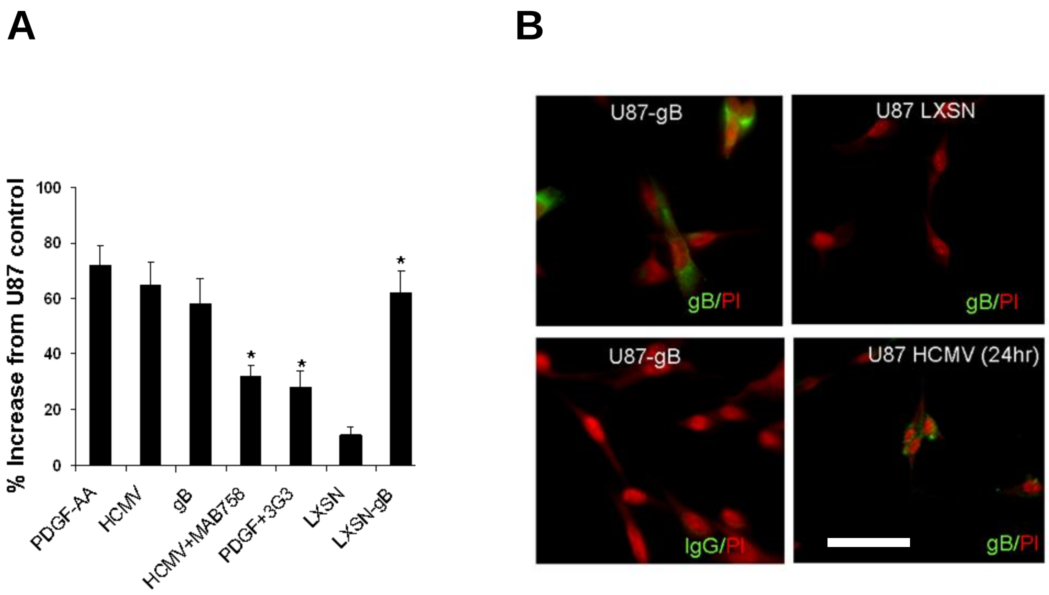

Supplementary Figure 3. A. LXSN-gB expressing glioma cells exhibit enhanced invasiveness. U87 parental cells and U87 cells transduced with retroviral control vector LXSN or LXSN-gB were subjected to a Matrigel invasion assay. Each condition was run in quadruplicate and the experiment was repeated twice. Representative results from one experiment are shown as percentage change from untreated U87 wild type cells. Student t Test was performed to assess significance.
*p=0.002 for HCMV+MAB758 vs HCMV. p=0.001 for PDGF+3G3 (PDGFRα blocking antibody) vs PDGF-AA and p=0.001 for LXSN-gB vs LXSN. **B**. U87 cells transduced with LXSN control or gB-expressing retroviruses were processed for immunofluorescence to detect gB (in conjunction with an anti-mouse Alexa 488 secondary antibody). Upper panels photomicrographs are showing the presence of gB on the plasma membrane of gB- transduced cells and not in the LXSN control cells. Lower left panel is a staining control, using an irrelevant matched IgG antibody and the bottom right panel shows the presence of gB in HCMV-infected (TR strain, MOI=1, 72h). Nuclei are counterstained with propidium iodide. Bar= 100μm.

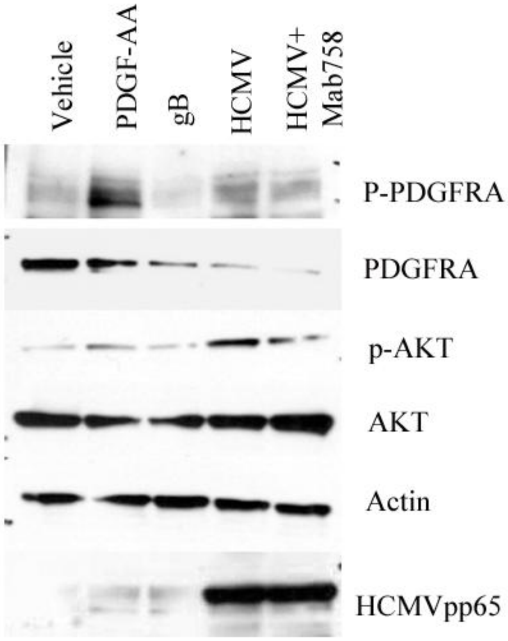

Supplementary Figure 4. HCMV gB activates the PDGFRα-p-AKT pathway in primary GBM cells. Primary-derived glioblastoma cells (line 4121) stimulated for 20 minutes as shown (PDGFAA, 20ng/ml, gB 50ug/ml, HCMV TR strain , MOI=1, MAB758 used at 10ug/ml) were used to generate lysates which were resolved on SDS-PAGE and probed with the indicated antibodies. HCMV as well as recombinant gB induced pAKT to levels comparable with the genuine ligand PDGF-AA. HCMV pp65 blot was used to demonstrate infection limited to the HCMV treated samples. The actin blot demonstrates equivalent loading.

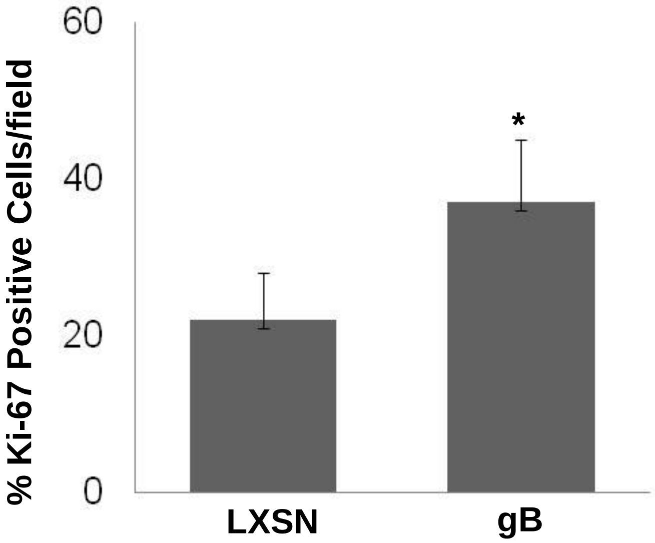

Supplementary Figure 5. Ectopic gB expression enhances tumor cell proliferation in glioma xenografts. Ki-67 immunohistochemical analysis (displayed in Figure 6) was quantified using 6 fields/ tissue slide. All tumor bearing animal xenograft tissues were used for quantification (n=12, in each group). *p=0.02 student t-Test.
